# The role of thrombectomy and diffusion-weighted imaging with MRI in post-transplant renal vein thrombosis: a case report

**DOI:** 10.1186/s12882-017-0618-2

**Published:** 2017-07-10

**Authors:** Paraish Misra, Anish Kirpalani, General Leung, Paraskevi A. Vlachou, Jason Y. Lee, Serge Jothy, Jeffrey Zaltzman, Darren A. Yuen

**Affiliations:** 1 Division of Nephrology, St. Michael’s Hospital, University of Toronto, Toronto, ON M5B 1W8 Canada; 2 Department of Medical Imaging, St. Michael’s Hospital, University of Toronto, 30 Bond Street, 3 Cardinal Carter South, Toronto, ON M5B 1W8 Canada; 3 Division of Urology, Department of Surgery, St. Michael’s Hospital, University of Toronto, 61 Queen Street East Suite 2-012, Toronto, ON M5C 2T2 Canada; 4 Department of Laboratory Medicine, St. Michael’s Hospital, 30 Bond Street, Room 2-015 CC Wing, Toronto, ON M5B 1W8 Canada; 5 Keenan Research Centre for Biomedical Science, Li Ka Shing Knowledge Institute, St. Michael’s Hospital, Rm 509, 5th Floor, 209 Victoria Street, Toronto, ON M5B 1T8 Canada

**Keywords:** Renal vein thrombosis, Kidney transplantation, Delayed graft function, Diffusion weighted magnetic resonance imaging, Case report

## Abstract

**Background:**

Surgical thrombectomy in the context of acute renal vein thrombosis (RVT) post-transplantation has had limited success, with considerable variation in the surgical techniques used. Unfortunately, it is usually followed by allograft nephrectomy within a few days if rapid allograft recovery does not ensue. We report a case of acute RVT in which nephrectomy was not performed despite a prolonged requirement for dialysis post-thrombectomy, but with recovery of renal function 2 weeks later. We also report the findings of serial MRI with diffusion-weighted imaging (DW-MRI) throughout the patient’s recovery, which provided novel insights into allograft microvascular perfusion changes post-thrombectomy.

**Case presentation:**

A 65-year old patient underwent living-unrelated kidney transplantation complicated by acute RVT. Surgical thrombectomy and irrigation led to a delayed, but significant, recovery of renal function. Serial non-contrast DW-MRI scanning was used to non-invasively assess microvascular renal blood flow post-operatively. Unlike standard Doppler ultrasonography, DW-MRI documented reduced microvascular perfusion initially, with gradual but incomplete recovery that mirrored the partial improvement in renal function.

**Conclusions:**

Our findings suggest that surgical thrombectomy may be more effective than previously described if followed by careful patient observation. Moreover, diffusion-weighted MRI appears to provide important insights into the pathophysiology of delayed graft function and deserves further investigation.

## Background

Renal vein thrombosis (RVT) is a dire complication of renal transplantation, leading to delayed graft function (DGF) and even graft failure. Although case series have described incidence rates of only 0.1–6%, RVT accounts for 21–78% of never-functioning allografts [1, 2]. RVT occurring early post-transplant (within 2 weeks) appears to confer a worse prognosis (2,15,16). Although occasional case reports have demonstrated that early RVT can be successfully managed with open thrombectomy [1–4], the vast majority of reported thrombectomy cases have ultimately required nephrectomy.

A key consequence of RVT is reduced microvascular perfusion, which is a phenomenon that also occurs in the much more common problem of DGF due to severe ischemia-reperfusion injury [5]. Regardless of the inciting cause, impairment of microvascular blood flow results in diminished oxygen delivery and ischemic injury, which can delay recovery, and if severe enough can lead to cortical necrosis. Currently, physicians have limited tools to non-invasively assess the severity of ischemic injury in cases of DGF due to RVT or ischemia-reperfusion injury. Doppler ultrasonography with resistive indices is the standard method for evaluating allograft health in many centres [6], despite it being shown to be largely ineffective for this purpose [7]. Meanwhile, acute kidney injury biomarkers such as KIM-1 correlate with severity of injury and possibly even mortality [8], but unfortunately they are not readily available, do not distinguish between injury in the allograft versus injury in the native kidneys, and provide no information on allograft microvascular perfusion changes or distribution of damage.

Advances in magnetic resonance imaging (MRI) have enabled the non-invasive measurement of microvascular blood flow without the need for gadolinium contrast. One such method, called diffusion-weighted MRI (DW-MRI), measures the movement of water molecules in tissues, and is routinely used to identify microvascular perfusion deficits in the setting of acute brain ischemia [9]. When combined with a mathematical post-acquisition technique called intravoxel incoherent motion (IVIM), microvascular perfusion can be quantified as the perfusion (“f”) fraction [10]. Consistent with the known perfusion impairment that occurs following ischemia-reperfusion injury [5], DW-MRI scanning has demonstrated reduced microvascular flow in transplant kidneys with DGF as compared to those with initial graft function [11–15]. To our knowledge, however, the natural history of changes in water mobility has never been explored following an episode of DGF. Moreover, DW-MRI scanning has never been studied in the context of acute RVT post-transplantation. Here, we describe a case of RVT post-transplant in which renal vein thrombectomy was initially followed by poor allograft function, followed by a delayed and unexpected renal recovery. We further present the progression of serial DW-MRI scan findings in this unusual case.

## Case presentation

**Fig. 1:**
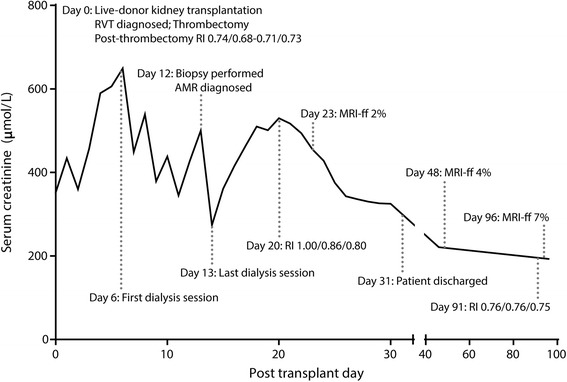
Timeline of events plotted against a graph of creatinine trajectory over time. *Resistive indices* are presented as upper pole/inter-polar region/lower pole. The *x axis* has been scaled to highlight the greater density of events during the patient’s admission (days 0–31). RVT, renal vein thrombosis. AMR, antibody-mediated rejection. RI, resistive index. MRI-ff, diffusion-weighted magnetic resonance imaging “f” fraction

A 65-year-old patient with end-stage renal failure on hemodialysis due to recurrent pyelonephritis was admitted to St. Michael’s Hospital for a living unrelated donor kidney transplant (see Fig. 1). Her residual native kidney urine output was less than 200 mL daily. An immunosuppression induction protocol of high-dose solumedrol and basiliximab was given pre-operatively. The procured allograft was the donor’s left kidney. Intra-operatively, the right external iliac artery was noted to be situated directly over the external iliac vein, causing a mild compression of the donor renal vein after anastomosis, without evidence of obstruction. Prior to closure, the renal vein was noted to be soft and patent, and allograft urine production was noted.

**Fig. 2:**
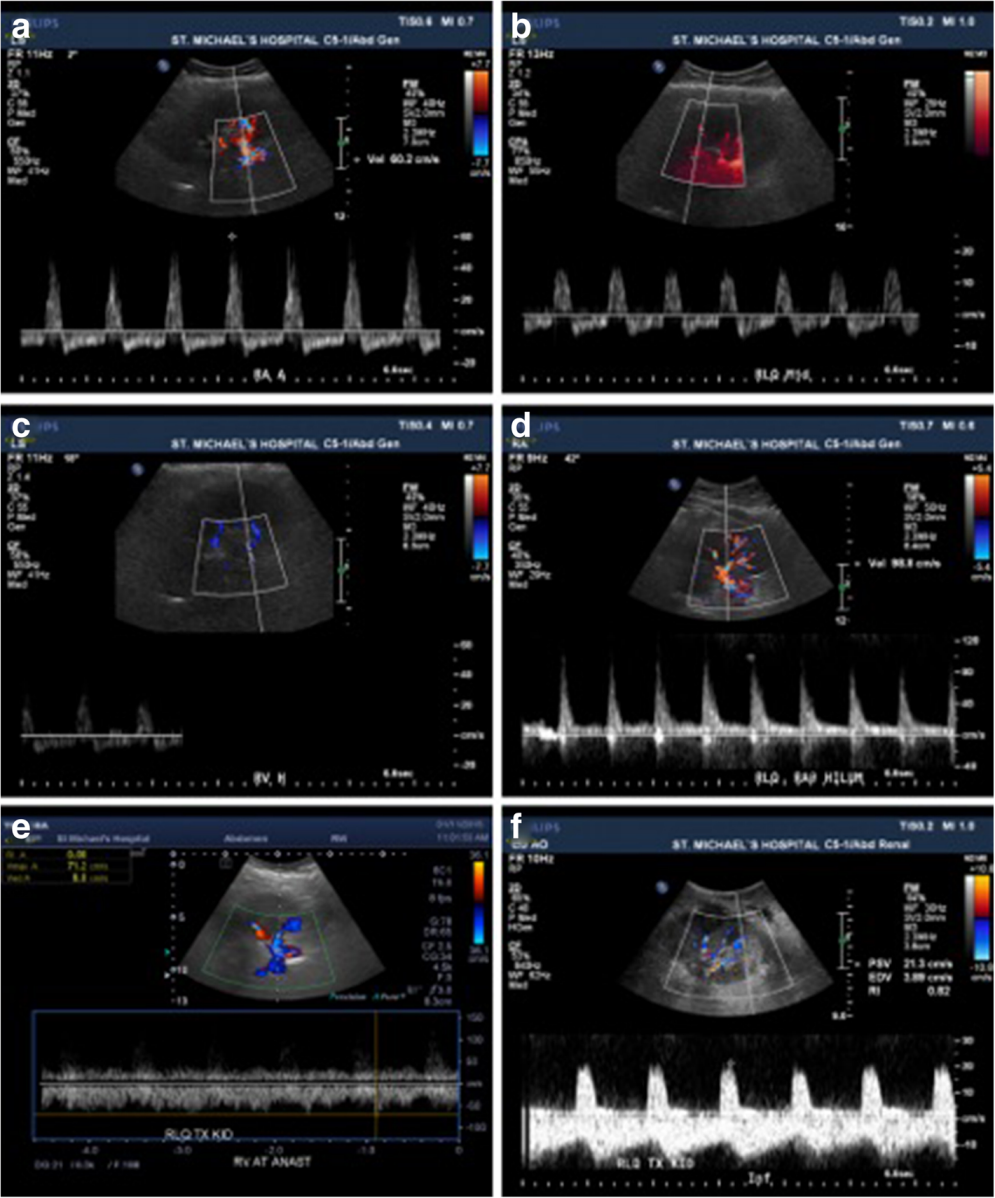
Representative images of serial Doppler ultrasound exams of patient with renal vein thrombosis post-kidney transplantation. Initial post-operative ultrasound showing (**a**) spectral tracing of reversed diastolic flow in the transplant renal artery near its anastomosis with color Doppler showing turbulent flow, (**b**) reversed intra-renal artery diastolic flow, and (**c**) difficulty finding transplant renal vein flow. Following successful thrombectomy, an ultrasound with color Doppler showed (**d**) normalized diastolic flow in the transplant renal artery and (**e**) patency of the transplant renal vein with normal venous waveform using spectral Doppler tracing. (**f**) On post-operative day 20, despite ongoing poor renal function, a Doppler ultrasound revealed relatively normal waveforms and calculated resistive indices that were not in keeping with the histology seen on biopsy (see Table 1 for resistive indices)

The patient was brought to the recovery room and remained hemodynamically stable, but urine output rapidly decreased over the ensuing 2 h. An ultrasound was performed immediately, which suggested acute RVT (see Fig. 2a – c). The patient was initiated immediately on intravenous heparin and returned to the operating room. The allograft was found to have large areas of mottled ischaemic parenchyma with small interspersed areas of well-perfused tissue. The venous and arterial anastomoses were taken down, at which time a large renal vein clot was noted. Following renal vein thrombectomy, the allograft was placed on ice-slush and immediately perfused with 2 l of cooled (4 °C), heparinised Custodiol HTK™ solution followed by 1 l of 4 °C normal saline over a 20 min interval. This aggressive irrigation led to flushing out of tiny micro-clots, such that following this procedure, the parenchyma was found to be mostly well-perfused (blanched) with only 5% of the visible kidney still purple and mottled.

At this point, allograft nephrectomy was contemplated, but the kidney was ultimately re-implanted for several reasons: (1) areas of pink, well-perfused parenchyma had been noted upon initial exploration, (2) the majority of the kidney appeared well-perfused after irrigation, (3) good low-pressure flows were noted through the graft, and (4) the allograft was from a living donor. Following re-anastomosis, the allograft renal vein was noted to be patent without compression by the renal artery, and as such the risk for recurrent renal vein thrombosis was felt to be significantly reduced.

Post-operatively, the patient continued to receive intravenous unfractionated heparin for 48 h. Due to a drop in hemoglobin from 90 g/L pre-operatively to 58 g/L on post-operative day (POD) 2, intravenous heparin was held. Serial allograft ultrasound scans were performed within the first post-operative week, which revealed no peri-nephric hematoma and normal resistive indices (RIs) (see Table 1 and Fig. 2d – e). The renal vein was found to be patent, and given that the presumed cause of the RVT (compression from the overlying renal artery) had been corrected, intravenous heparin was not resumed. The patient was initiated on prophylactic low-dose subcutaneous heparin on POD 6. Maintenance immunosuppression, which had been initiated on POD1, consisted of prednisone, long-acting tacrolimus and mycophenolate, and was never interrupted throughout the post-operative course.

**Table 1:** Representative clinical and Doppler ultrasound imaging parameters

Post-operative day (#)	Serum Creatinine (μmol/L)	Resistive Indices	Notes
0	352	Upper pole: 0.8	Pre-thrombectomy
		Interpolar: 0.88	
		Lower pole: 0.83	
1	434	Upper pole: 0.74	Post-thrombectomy
		Interpolar: 0.68–0.71	
		Lower pole: 0.73	
3	457	Upper pole: 0.77	
		Interpolar: 0.74	
		Lower pole: 0.73	
4	590	Upper pole: 0.78	
		Interpolar: 0.79	
		Lower pole: 0.79	
6	649		Dialysis performed
7	449	Upper pole: 0.81	
		Interpolar: 0.82	
		Lower pole: 0.80	
8	539		Dialysis performed
9	379	Upper pole: 0.80	
		Interpolar: 0.83	
		Lower pole: 0.80	
10	438		Dialysis performed
12	427		Biopsy performed
13	500		Last dialysis session
14	274	Upper pole: 0.93	
		Interpolar: 0.87	
		Lower pole: 0.87	
15	361		
17	463		
18	510	Upper 0.83	
		Inter 0.87	
		Lower 0.85	
19	501		
20	530	Upper 1.0	
		Inter 0.86	
		Lower 0.8	
23	455		DW-MRI “f” fraction 2%
30	325		
46	221		
48	N/A		DW-MRI “f” fraction 4%
91	N/A	Upper: 0.76	
		Inter: 0.76	
		Lower pole: 0.75	
96	193		DW-MRI “f” fraction 7%

**Fig. 3:**
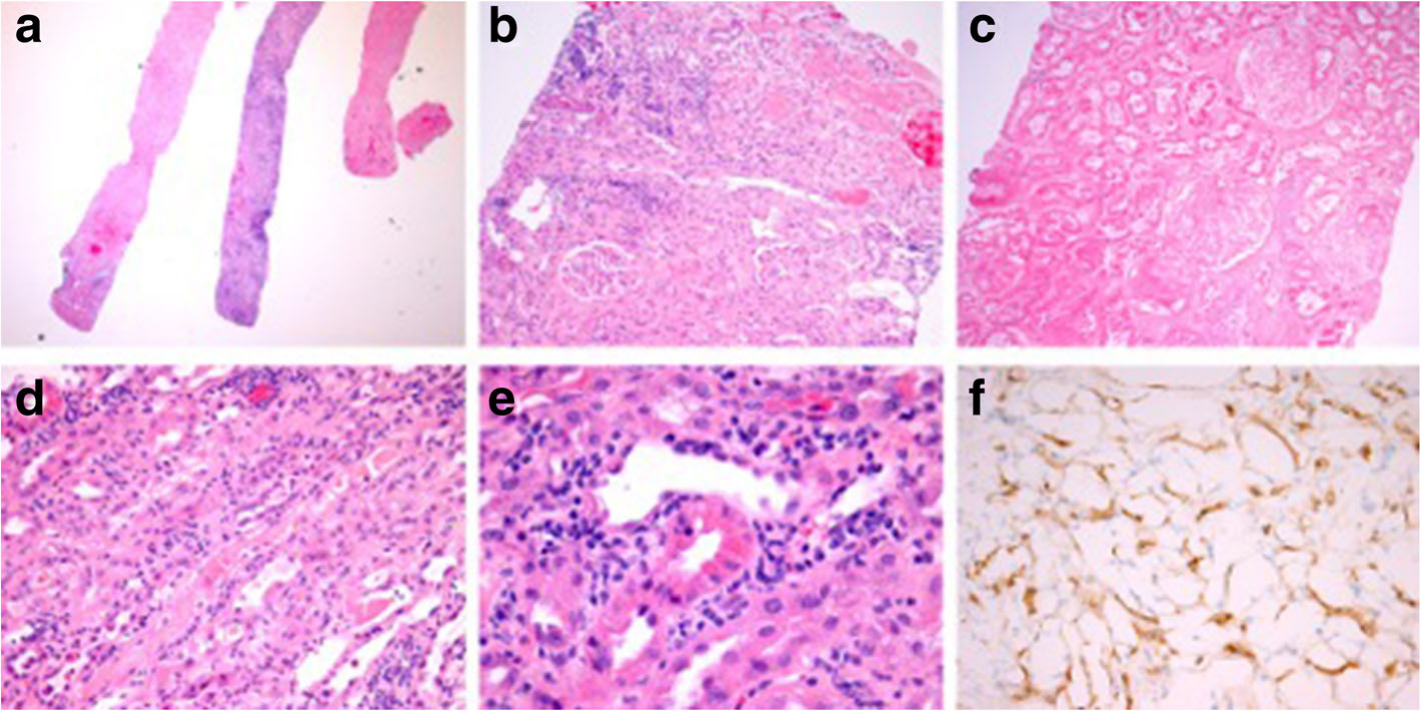
Representative images of kidney biopsy performed on post-operative day 12. (**a**) A low power image of three of the five core biopsies, with two of the visualized cores demonstrating extensive cortical necrosis. Original magnification 12.5X. (**b** – **c**) Higher power images of areas of cortical necrosis, with evidence of complete destruction of glomerular and tubular structures, and associated inflammatory infiltrates. Original magnification 100X. Areas of preserved cortex demonstrated (**d**) capillaritis and interstitial inflammation, (**e**) tubulitis, and (**f**) peritubular capillary C4d staining. Original magnification 400X

**Fig. 4:**
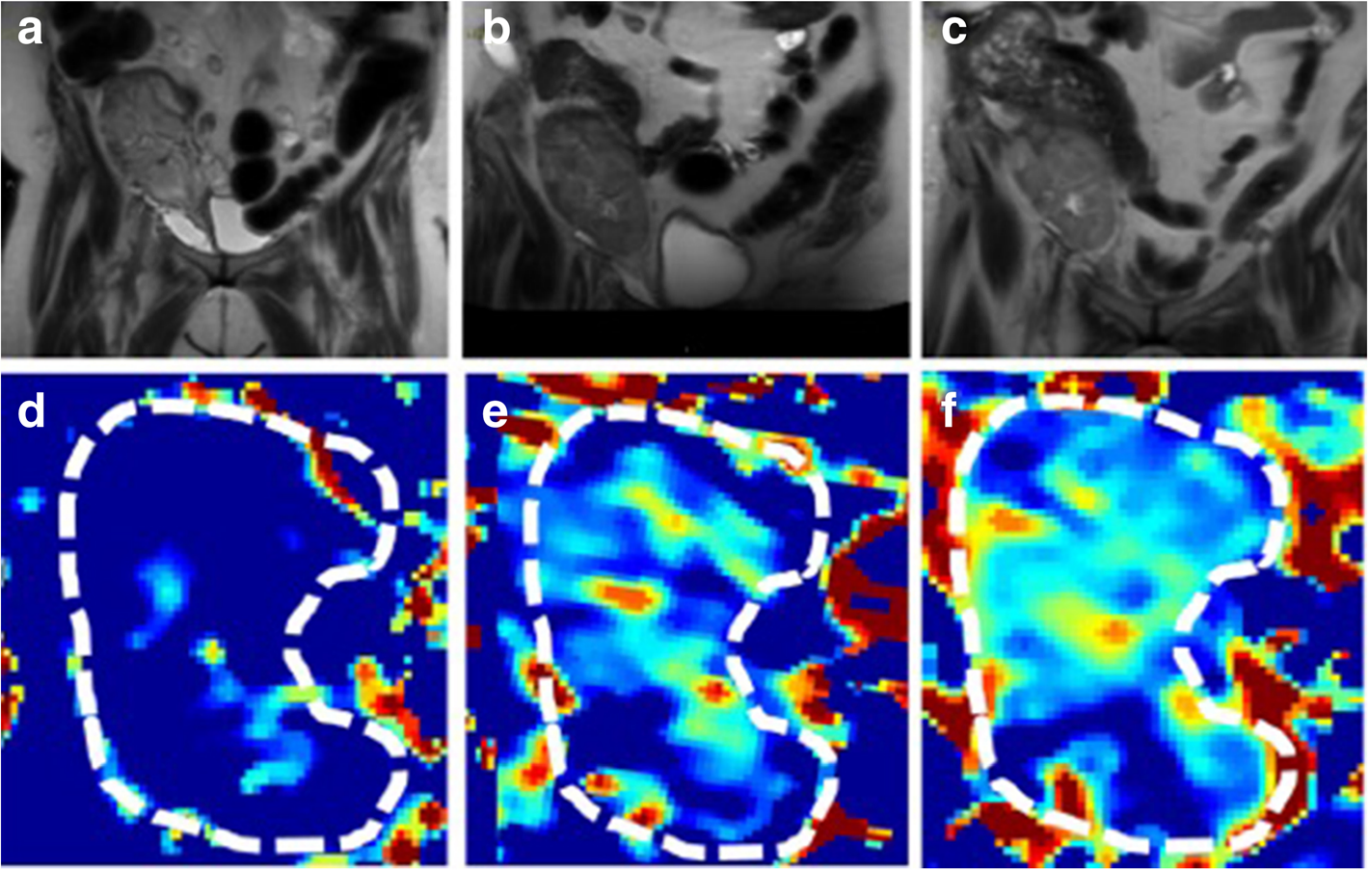
Serial diffusion-weighted MRI scans demonstrated improvements in allograft perfusion following transplant renal vein thrombectomy. (**a** – **c**) Serial conventional T2 weighted images, with grossly abnormal high T2 signal throughout the allograft parenchyma. (**a**) POD 22 scan. (**b**) POD 48 scan. (**c**) Scan at 3 months post-transplant vein thrombectomy. (**d** – **e**) Serial pseudocolorized perfusion (“f” fraction) maps derived from diffusion-weighted MRI images, progressing from (**d**) minimal perfusion on POD 22, to improved but still abnormally low perfusion (**e**) on POD 48 and (**f**) at 3 months post-thrombectomy. The *color bar* depicts the magnitude of the perfusion fraction, with *red* indicating higher perfusion, and *blue* indicating lower perfusion

Daily urine output increased progressively over the first 5 days post-thrombectomy, and then fluctuated between 175 mL and 600 mL over the following 10 days. Combined with the encouraging Doppler ultrasound scans during this time (see Table 1), it was felt that delaying a biopsy of the allograft was reasonable despite the initiation of dialysis on POD 6. On POD 11, however, urine output decreased to 100 mL over 24 h, resulting in a biopsy on POD 12. Of the five cores sampled, three showed extensive cortical necrosis with complete destruction of glomerular and tubular structures (see Fig. 3a – c). The remaining viable tissue demonstrated tubulitis, interstitial inflammation, endarteritis, and positive peritubular capillary C4d staining (see Fig. 3d – f). A diagnosis of cortical necrosis with associated acute antibody-mediated rejection was made, and the patient was treated with pulse intravenous corticosteroids and intravenous immunoglobulin at a dose of 1 mg/kg daily for 3 days. Dialysis was held after POD 13 to assess for renal recovery, but the creatinine continued to increase, and an ultrasound was repeated on POD 20. This demonstrated RIs mostly between 0.80 and 0.85, but with values up to 1 at the superior pole (see Table 1, Fig. 2f). To further characterize renal microperfusion, a non-contrast DW-MRI was performed. The perfusion “f” fraction was found to be 2% with diffusely distributed patches of reduced microvascular flow (see Fig. 4a, d). It is our experience (data not shown) and that of others that MRI scans of transplant kidneys with normal function reveal higher perfusion “f” fractions of >10% [16].

Over the ensuing days, the patient’s urine output increased, and no further dialysis sessions were required (see Fig. 1). The patient was ultimately discharged on POD 31 with a creatinine of 327 μmol/L. On POD 48, the serum creatinine was 221 μmol/L, and repeat DW–MRI showed an increased perfusion “f” fraction of 5%, again with patches of reduced microvascular flow (see Fig. 4b, e). In contrast, a Doppler ultrasound was essentially normal, showing a patent renal vein and RIs of 0.75–0.76 (see Table 1). Three months post-transplant, the patient remained dialysis-free with a serum creatinine of 194 μmol/L, with a final DW-MRI scan at this time point showing a “f” fraction of 7% with patchy reductions in microvascular perfusion (see Fig. 4c, f).

## Discussion and conclusions

We discuss a case of early RVT post-transplantation with delayed recovery after immediate open thrombectomy. RVT is generally a catastrophic complication of kidney transplantation, often managed with immediate allograft nephrectomy [1, 2, 17, 18]. Published reports describing cases managed with thrombectomy usually give little information regarding the intra-operative findings or the procedure performed, and often do not detail the duration of oligoanuria post-thrombectomy after which the allograft was deemed non-salvageable [1, 19, 20]. Of the three case reports describing successful thrombectomy that also describe the post-operative course, all three patients were independent from dialysis within 1 week [4, 21]. Our patient demonstrated remarkable renal reserve and regenerative capacity, eventually gaining independence from dialysis 2 weeks post-thrombectomy despite prolonged oliguria and the development of antibody-mediated rejection. We speculate that if a good outcome could be obtained with such a severely affected allograft, other cases may also demonstrate improvement if early intervention is followed by careful patient observation. However, the duration of observation prior to declaration of irreversible failure requiring nephrectomy remains unclear, and novel modalities for monitoring allograft health in this setting are clearly needed.

Successful salvage of a renal allograft affected by RVT may be contingent on surgical technique. As no standardized approach exists, surgical intervention varies according to surgeon experience. In our case, thrombectomy was followed by prolonged cold irrigation, which in the limited series of reports published to date, has usually led to good outcomes [3, 4, 20]. Irrigation fluid composition may further influence the likelihood of success. Such solutions include nutrient-rich fluids such as Custodiol HTK™, or mixtures of heparin and vasodilators [3], but the optimal composition remains unknown.

Despite its importance in the pathogenesis of DGF, clinicians have few tests available for the evaluation of allograft microvascular perfusion. Aside from biopsy, Doppler ultrasound is generally the only other standard test for the evaluation of DGF at our centre and others [7]. The main benefit of Doppler ultrasound imaging is its ability to examine the patency of the allograft macrovessels and collection system. However, Doppler ultrasound is also commonly used to calculate intra-renal resistive indices, as a measure of microvascular blood flow. In our case, ultrasound imaging did establish the presence of renal vein thrombosis, but as in most cases, its utility in evaluating the severity of microvascular injury was quite limited. Indeed, a recent report found no correlation between RIs and clinically meaningful outcomes such as allograft histology or the need for dialysis, but rather correlated with recipient factors such as recipient age and central hemodynamics [7]. In line with these findings, our patient had normal RIs immediately post-transplant despite the presence of extensive micro- and macro-vascular clot and reduced parenchymal perfusion, oliguria, and rising serum creatinine. By POD 20, the RIs remained only slightly elevated in the majority of the allograft (Fig. 2f), which were incongruent with allograft performance (the patient had minimal allograft function at that time). In contrast, a DW-MRI performed on POD 22 demonstrated markedly reduced intra-renal microvascular perfusion (see Fig. 4d), a finding more consistent with the residual microvascular clots seen intra-operatively, and the clinical picture at that time. Three months post-transplant, when the patient remained dialysis-independent but with significant renal dysfunction with a serum creatinine of 194 μmol/L (eGFR 23 mL/min), the Doppler ultrasound demonstrated normal RIs of 0.75–0.76, whereas DW-MRI showed improvements in blood flow on the background of persistent areas of impaired perfusion (see Fig. 4c, f). Given the importance of microvascular perfusion deficits in the pathogenesis of DGF due to RVT and severe ischemia-reperfusion injury, this case reinforces the lack of utility of ultrasound-based RI measurements for the evaluation of DGF, and highlights the potential of DW-MRI as a non-invasive means for gaining insights into DGF pathophysiology that are not readily apparent with other standard measures such as serum creatinine.

To our knowledge, we are the first to report the natural history of perfusion changes in a patient with DGF using DW-MRI, a finding that would not have been possible with standard measures such as biopsy (which can examine only structural but not functional changes), serum creatinine levels, or Doppler ultrasound. Three cross-sectional studies have described DW-MRI findings in patients with DGF, consistently demonstrating reduced perfusion when compared to controls with preserved renal function [11, 13, 15]. Taken together, our case, combined with these other studies, suggests that unlike Doppler ultrasound or other standard allograft measures such as serum creatinine or biopsy analysis, DW-MRI can evaluate graft microvascular perfusion, and therefore may be a useful non-invasive and contrast-free way to follow a key contributor to DGF pathogenesis. As such, although our data is limited to a single patient with a rare cause of DGF and must be interpreted in that context, this report provides an early rationale for further investigating the role of DW-MRI as a measure of allograft microvascular perfusion changes in patients with DGF.

In summary, we provide details of an unusual case of delayed renal recovery following successful management of RVT post-living donor kidney transplantation. We also describe the use of serial DW-MRI scans as a novel means to image the impaired microvascular perfusion associated with a rare cause of DGF, and the subsequent increase in microvascular flow as renal function improved. In contrast, standard Doppler ultrasound imaging did not correlate well with either histology or the patient’s clinical presentation. As impairment of microvascular blood flow is a critical contributor to most forms of DGF, future work studying the utility of DW-MRI will be helpful in defining its role in the evaluation of DGF due to RVT and other causes.

## Abbreviations

DGF: Delayed graft function
DW-MRI: Diffusion-weighted magnetic resonance imaging
IVIM: Intravoxel incoherent motion
MRI: Magnetic resonance imaging
POD: Post-operative day
RVT: Renal vein thrombosis
